# Effectiveness and User Experience of Immersive Virtual Reality in Cognitive Rehabilitation for Attention-Deficit/Hyperactivity Disorder: Systematic Review

**DOI:** 10.2196/71963

**Published:** 2025-12-12

**Authors:** Natalie Single, Winona Mishelle Graham, Joshua Kelson, Danielle Sulikowski

**Affiliations:** 1 School of Psychology, Faculty of Business, Justice and Behavioural Sciences, Charles Sturt University, Panorama Avenue, Bathurst, NSW, 2795, Australia, 61 2 6338 4570; 2 School of Psychology, Faculty of Business, Justice and Behavioural Sciences, Charles Sturt University, Port Macquarie, NSW, Australia

**Keywords:** attention-deficit/hyperactivity disorder, ADHD, virtual reality, VR, cognitive rehabilitation, cognitive training, rehabilitation, remediation, efficacy, effectiveness, user experience, safety, usability, acceptability, memory, attention, hyperactivity, inhibitory control, systematic, review method, review methodology, psychiatric, mental health

## Abstract

**Background:**

Attention-deficit/hyperactivity disorder (ADHD) is a neurodevelopmental disorder characterized by difficulties in attention, impulsivity, and hyperactivity. These difficulties can result in pervasive and longstanding psychological distress and social, academic, and occupational impairments.

**Objective:**

This systematic review aims to investigate the effectiveness and user experience (ie, safety, usability, acceptability, and attrition) outcomes of immersive virtual reality (VR) interventions for cognitive rehabilitation in people with ADHD and identify research gaps and avenues for future research in this domain.

**Methods:**

Peer-reviewed journal articles that appraised the treatment impact of any immersive VR-based intervention on cognitive abilities in people of all ages with ADHD were eligible for inclusion. The following databases were searched up until November 2024: Cochrane Library, IEEE Explore Digital Library, PsycINFO, PubMed, Scopus, and Web of Science. Records were screened on title and abstract information after deduplication, leading to full-text appraisal of the remaining records. Findings from eligible articles were extracted into a standardized coding sheet before being tabulated and reported with a narrative synthesis.

**Results:**

Out of 1046 records identified, 15 articles met the inclusion criteria. Immersive VR-based interventions for people with ADHD were generally effective in improving cognitive abilities, such as attention, memory, and executive functioning. User experience outcomes were also generally positive, with low levels of simulator sickness and minimal attrition reported during VR-based treatment.

**Conclusions:**

Immersive VR-based interventions hold promise for effectively, safely, and rapidly treating cognitive deficits in children and adults with ADHD. However, more studies are required to examine their longitudinal impact beyond treatment cessation.

## Introduction

### Background

Attention-deficit/hyperactivity disorder (ADHD) is a prevalent neurodevelopmental disorder characterized by persistent difficulties with attention, impulsivity, and hyperactivity [1,2]. ADHD predicts psychosocial and occupational dysfunction, and increased health care and education costs [3]. Common treatments for ADHD include pharmacotherapy, behavior therapy, cognitive behavior therapy, behavioral teacher and parent training, organizational and social skills training, lifestyle change, or combination therapies [4-6]. These treatments can assist with cognitive rehabilitation, which aims to improve functional deficits in cognitive abilities, such as perception, attention, memory, and executive functions [7]. In recent years, innovations in immersive virtual reality (VR) technology have been integrated into cognitive rehabilitation treatments for ADHD [8].

Immersive VR commonly uses a computerized head-mounted display (HMD) to obscure the external environment and immerse individuals in a digital environment [9]. In contrast, nonimmersive VR provides digital environments displayed on monitors or projected onto screens, which do not obscure the surrounding environment. Interacting in nonimmersive environments is typically mediated by mouse, keyboard, or touchscreen [10]. Immersive VR technology holds greater promise for procuring therapeutic gains than nonimmersive VR due to its capacity to induce presence—the sense of being physically present in the digital environment [10]. For example, greater presence has been linked to enhanced memory performance in immersive VR [11], but not in nonimmersive VR interventions [12]. This may be because the heightened sense of presence in immersive VR is associated with increased enjoyment, emotional engagement, and attention; factors that can support information processing and retention, although findings remain mixed [13,14].

Immersive VR flexibly delivers cognitive rehabilitation treatment for ADHD [8,15-19]. For instance, HMDs can deploy customizable VR environments that contain distracting stimuli, simulated social interactions, and tasks that require multitasking in common settings such as work, home, and school [20-22]. Individuals with ADHD can practice their skills for focusing and coping in these controlled digital environments [15,16]. Immersive VR can minimize exposure to uncontrolled external distractions that interfere with focus, while also reducing reliance on inconsistent or delayed reinforcers, such as praise or outcomes contingent on facilitator availability [14,23,24]. Instead, it can incorporate strategically designed and controlled distractors [25,26] along with structured, immediate rewards, such as points or visual effects, to strengthen cognitive abilities through targeted training [27,28].

Immersive VR training experiences complement other cognitive rehabilitation treatments, such as pharmacotherapy and cognitive behavior therapy [8,19,29]. However, immersive VR may be uniquely beneficial compared with traditional treatments. For example, VR-based biofeedback has outperformed standard 2D hemoencephalographic biofeedback in treating attention deficits in children with ADHD [26]. The effectiveness of VR likely stems from its ability to heighten motivation and engagement during cognitive rehabilitation training [25,26,30,31]. Increased motivation may result from immersion [26], novelty in the virtual environment [12], or gamification elements such as material rewards (eg, coins, tokens) [27].

The motivational features of VR not only increase engagement but may also stimulate dopaminergic activity in brain regions involved in memory and executive functions [12,27,31]. This is particularly beneficial for children with ADHD who have dopamine deficits that contribute to impairments in cognitive functioning [12,27,31,32]. For example, children with ADHD typically show weaker memory encoding due to low dopamine levels in the substantia nigra and ventral tegmental area, midbrain structures that help regulate dopamine release into the hippocampus [12,33]. However, exposure to novel digital environments during a critical postlearning period can enhance dopamine release, triggering protein synthesis necessary to stabilize and consolidate memory traces [12].

Similarly, material rewards delivered through VR can stimulate dopamine release in the brain’s reward system [27]. Dopaminergic activity reinforces behavior by increasing motivation and enhancing prefrontal cortex activity, thereby supporting executive functions such as attention regulation and inhibitory control, which are essential for suppressing inappropriate actions and facilitating goal-directed behavior [27]. These mechanisms help explain how immersive VR uniquely engages dopaminergic and executive function networks impaired in ADHD, offering a neurobiologically grounded rationale for its therapeutic potential. VR-based interventions may also drive neuroplastic changes by repeatedly engaging neural networks involved in attention, inhibition, and working memory over time, facilitating functional reorganization in these circuits [25,28]. Thus, immersive VR may activate and strengthen underlying neural pathways disrupted in ADHD, supporting cognitive change.

VR-based interventions can improve cognitive deficits among individuals with ADHD [8,17,18,29,34]. For example, samples of ADHD-diagnosed children exposed to VR-based interventions show improved attention from pretest to posttest, with Hedges *g* of 0.09 to 1.21 [35] and *g*=1.46 [36]. Similar benefits of VR-based interventions are reported when compared against empty control groups, *g*=1.77 [36], and a psychotherapy intervention, *g*=0.57 to 1.4 [35], although not compared with pharmacological treatment, *g*=–0.28 to −0.31 [35]. In the same population, similar improvements in impulsivity are observed after VR-based interventions, *g*=0.48 to 0.91 [35], and *g*=1.77 [36]. Such improvements exceed empty control groups, *g*=1.27 [36], and both psychotherapy intervention, *g*=0.46 to 0.52, and pharmacological treatment, *g*=0.8 to 0.81 [35]. VR-based intervention combined with biofeedback decreases impulsivity, *g*=2.31, and increases attention, *g*=1.21 to 1.85, pretest to posttest [26]. For both impulsivity (*g*=0.77 to 0.82) and attention (*g*=0.66 to 1.05), gains exceed those of biofeedback alone [26]. VR-based interventions also increase memory function pretest to posttest *g*=0.8 to 1.56 [33,37], exceeding both an empty control group, *g*=3.36, and pharmacological treatment, *g*=1.27 [33]. Hence, VR-based interventions can improve attention, impulsivity, and memory in children diagnosed with ADHD, and in some cases, do so as effectively, or more effectively than psychotherapy, pharmacological treatment, and biofeedback.

Despite emerging positive effectiveness outcomes for cognitive rehabilitation with VR-based interventions, this technology may not be appropriate for all people with ADHD [8,38]. For instance, the financial cost of VR equipment may be prohibitive for some clinicians and individuals [39]. Some individuals may experience safety issues with immersive VR technology, such as physical collisions with real-world objects and simulator sickness, which includes symptoms of nausea, fatigue, eye strain, blurred vision, headache, dizziness, and vertigo [40-42]. Emotion dysregulation may also occur during the use of VR. This may include dissociation, panic, and anxiety, posing problems for safe containment, particularly if individuals use VR in a self-help format without the supervision and support of a trained clinician to assist them [43]. Some people may also experience usability issues when interacting with new technology, such as finding it hard to learn, being inefficient in performing tasks, being prone to errors, being unmemorable, and being dissatisfying to use [44]. These issues can impact user acceptability, which refers to the willingness of a person to use a VR intervention [45]. If a user finds an intervention unacceptable, they may reject using it, which can lead to attrition within research studies and poorer therapeutic outcomes [46].

### This Study

Available research broadly supports the effectiveness of VR-based treatments for ADHD. However, the scope of previous reviews has tended to include nonimmersive VR intervention types, while being limited by treatment population (eg, focusing solely on children with ADHD), study design (eg, limiting to randomized controlled trials [RCTs] only), and measured outcomes (eg, focus on effectiveness outcomes rather than accompanying user experience outcomes) [8,15,16,19,29]. This exposes a deficit of collated knowledge on the effectiveness and user experience of immersive VR-based treatments for cognitive rehabilitation of people of all ages with ADHD. Therefore, a systematic review was conducted to (1) comprehensively explore all research designs conducted in the use of immersive VR in the cognitive rehabilitation for people of all demographics with ADHD, (2) to establish the full range of cognitive rehabilitation interventions being used with immersive VR, (3) report on concomitant outcomes of effectiveness and user experience (ie, safety, acceptability, usability, and attrition), and (4) identify research gaps and make suggestions that can inform clinical practice and future research in this domain.

## Methods

### Overview

The PRISMA (Preferred Reporting Items for Systematic Reviews and Meta-Analyses) method was adopted for this systematic review (Checklist 1) [47]. The review protocol was not registered.

### Eligibility Criteria

To be eligible for inclusion in this review, studies needed to be written in English; published in a peer-reviewed journal article; to have examined the treatment effects of any immersive VR-based intervention (ie, using a HMD or cave automatic virtual environment) on any cognitive ability; and have a sample comprised mostly of participants with ADHD (ie, 80% or above proportion) or reported subset analyses for participants with ADHD. All comparators and study designs were eligible for inclusion. No gray literature was included in this review, and no studies were excluded based on quality appraisal.

### Search Strategy

The following databases were searched up until November 2024: Cochrane Library, IEEE Explore Digital Library, PsycINFO, PubMED, Scopus, and Web of Science. Search strategy keywords included: (“virtual reality” and (“ADHD” or “attention deficit” or “hyperactivity disorder”). A manual search was also conducted on the reference lists of included studies.

### Article Selection

Search results were extracted and deduplicated. Records were initially screened based on title and abstract information. Potential articles underwent full-text appraisal to determine whether they met the inclusion criteria. Differing views on study inclusion were resolved through discussion and consensus on inclusion by the full research team.

### Data Charting

Data extracted from included studies (by one author, NS) included full reference citation, participant details (country of origin, sample size, age, sex, and ADHD diagnosis), intervention details (name, aim, software, hardware, treatment focus, treatment length, and tasks), study details (methodology, measures, comparators, and measurement time points), and main findings (pertaining to effectiveness, safety, usability, acceptability, and attrition).

In this review, the acceptability of immersive VR is defined in terms of participants’ willingness to use, actual usage, and satisfaction after use of VR [46]. Usability in this review refers to how easy it is to learn, efficient, memorable, error-free, and subjectively pleasing the immersive VR experience is to the users [44]. Extracted data were tabulated and then checked by 2 other reviewers (DS and WG), and any divergent views on inclusion were resolved through discussion and agreement by the full research team.

### Quality Assessment

Included studies were critically appraised with the Mixed Methods Appraisal Tool [48]. With this tool, each study is subjected to 2 screening questions and then assigned to one of 5 study categories: qualitative research, RCT, nonrandomized, quantitative descriptive, or mixed methods. Each category has 5 questions that can be answered to appraise the quality of the studies.

### Data Analysis

Due to the required integration of qualitative and quantitative findings to answer our research questions, a narrative synthesis approach was adopted. This approach summarizes the findings in text and explains them in a descriptive manner.

## Results

### Study Selection

**Figure 1.**
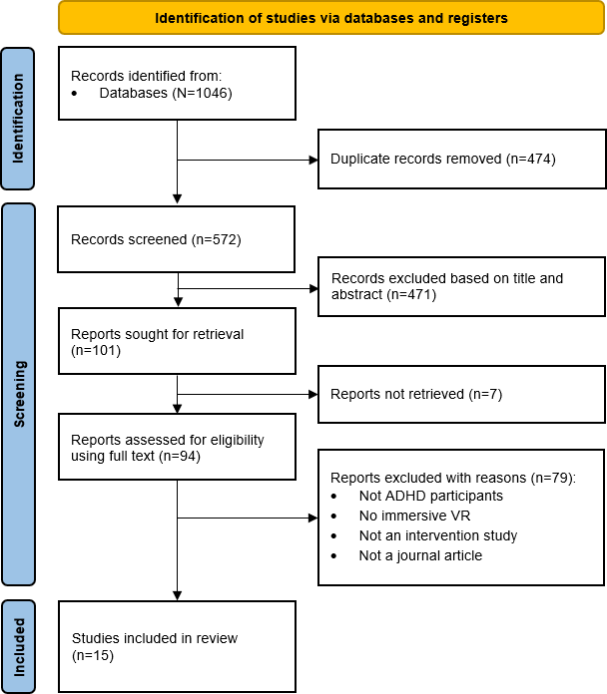
Flowchart of systematic review search results. ADHD: attention-deficit/hyperactivity disorder; VR: virtual reality.

Our search generated 1046 records (Figure 1). A total of 572 (54.7%) records remained after deduplication. Of these 572 records, 15 (2.6%) met the inclusion criteria for this review.

### Participant Characteristics of Included Studies

There were 754 participants across all included studies (Table 1). Participants primarily came from Italy (n=119), followed by Hong Kong (n=90), Spain (n=89), Poland (n=87), Greece (n=80), Romania (n=59), France (n=51), Iran (n=48), South Korea (n=40), Germany (n=36), United States (n=27), Portugal (n=25), and Taiwan (n=3). Sample sizes ranged from 3 to 90 participants with a median of 51 (IQR 25‐80). Participant ages ranged from 5 to 25 years. Participants were primarily boys and men (n=501). The average female sample proportion was 41.8% (SD 22.7%; range 16.1%‐96.0%). Most participants had diagnosed ADHD (n=703) or exhibited symptoms indicative of ADHD (n=25).

**Table 1.** Details on participants and research design of included studies.

Study	Country	Sample size^a^	Total sample age (years), mean (SD)	Total sample age range (years)	Sample (percentage) of female participants	Study design	Treatment conditions (N)^b^
Bioulac et al [35]	France	51	8.9 (1.2)	7‐11	10 (19.6)	RCT^c^	VR^d^ (16); Methylphenidate (16); Psychotherapy (19)
Cuber et al [49]	United States	27	21.0 (1.60)	18‐25	15 (55.6)	Mixed methods	VR (27)
Cunha et al [25]	Portugal	25	21.0 (0.85)	—^e^	24 (96.0)	RCT	VR (13); Passive control group (12)
David et al [50]	Romania	59	8.5 (1.6)	6‐11	12 (20.3)	RCT	VR with psychotherapy and atomoxetine (20); Atomoxetine (21); VR with psychotherapy (18)
De Luca et al [30]	Italy	59	8.0 (3.8)^f^	—	13 (22.0)	Quantitative nonrandomized	VR group (30); Traditional therapy group (29)
Kim et al [51]	South Korea	24	10.8 (1.7)	8‐13	14 (58.3)	Quantitative nonrandomized	Pulmonary VR training with psychomotor program (12); Psychomotor program (12)
Oh et al [52]	South Korea	16	8.75	8‐13	10 (62.5)	Quantitative nonrandomized	VR with ADHD^g^ (8); VR healthy controls (8)
Ou et al [32]	Taiwan	3	9.7	8‐12	2 (66.7)	Quantitative descriptive	VR (1); VR with medication (1); VR with medication and occupational therapy (1)
Rodrigo-Yanguas et al [53]	Spain	89	14.4 (2.3)	12‐22	33 (31.7)	RCT	VR serious game (31); Therapeutic chess (24); Control intervention (34)
Schena et al [54]	Italy	60	—	5‐12	27 (45.0)	RCT	VR (30); Speech and psychomotor therapy (30)
Selaskowski et al [31]	Germany	36	31.0 (7.9)	—	13 (36.1)	Quantitative nonrandomized	VR feedback, sham feedback, no feedback; ADHD group (18); Healthy control group (18)
Sergis et al [28]	Greece	80	25 (—)	—	36 (45.0)	Quantitative nonrandomized	ADHD Dog VR game (40); ADHD Dog conventional game (40)
Skalski et al [26]	Poland	87	12.75 (1.6)	9‐15	14 (16.1)	RCT	BF^h^ (30); VR-BFD^i^ (28); VR-BF^j^ (29)
Tabrizi et al [33]	Iran	48	9.6 (1.8)	7‐12	16 (33.3)	RCT	VR (16); Medication (16); No intervention control (16)
Wong et al [23]	Hong Kong	90	8.5 (1.7)	6‐12	17 (18.9)	RCT	VR-based social skills training (30); Conventional social skills training (30); Waitlist control (30)

a Refers to the number of participants in the final analyzed sample of the study.

b Refers to the number of participants in each treatment condition.

c RCT: randomized controlled trial.

d VR: virtual reality.

e Not available (not reported).

f Values provided in graphical form in original paper only.

g ADHD: attention-deficit/hyperactivity disorder.

h BF: standard hemoencephalographic biofeedback with physical activity training.

i VR-BFD: virtual reality and hemoencephalographic biofeedback with distractors.

j VR-BF: virtual reality and hemoencephalographic biofeedback without distractors.

### Details of the Virtual Reality Interventions

All included studies tested a unique VR-based intervention (Table 2). Immersive HMDs were used to host digital environments with a focus on treating various cognitive abilities. No studies used cave automatic virtual environment technology. Individual VR treatment session lengths varied from 3 to 60 minutes. Overall intervention length varied from a single session (50 min) to 6 months, with a median time of 7 weeks (IQR 4‐12 wk). The number of VR treatment sessions ranged from 1 to 36, with a median of 10 (IQR 8‐12). All VR-based interventions were tested with therapist or facilitator guidance.

**Table 2.** Findings on VR^a^ interventions and user engagement outcomes.

Study	Software	VR headset	Treatment focus	Treatment length
Bioulac et al [35]	CPT^b^ with audio and visual distractors in a virtual classroom environment.	—^c^	Attention and inhibitory control	12 sessions over 6 weeks; 30 min per session
Cuber et al [49]	Self-directed study in a virtual cabin environment	Varjo XR-3	Concentration, motivation, and effort on academic tasks.	Up to 12 (average of 7.4) sessions over 6 weeks; 50 min per session
Cunha et al [25]	Six games from Enhance VR, 3 games per session	Meta Quest HMD	Working memory and processing speed	10 sessions over 5 weeks; 30 min per session
David et al [50]	Black board attention task in a virtual classroom (Clinica VR Classroom).	—	ADHD^d^ primary symptoms and internalizing and externalizing symptoms	1 session (50 min), delivered within psychotherapy program of 16 weekly sessions
De Luca et al [30]	Serious games from the BRAVO Project	HTC Vive	Auditory and visual attention, working memory, inhibition, and control	6 months of game play (frequency and duration of sessions not reported)
Kim et al [51]	Gamification of feedback to respiratory input, ATA^e^	Samsung Gear VR	Visual attention and general pulmonary function	8 training sessions over 4 weeks, 15 min per session
Oh et al [52]	A VR outdoor roller coaster scenario based on the ATA and the Stroop Color-Word Test.	Samsung Odyssey VR HMD	Attention and inhibitory control	8 sessions over 8 weeks; duration of sessions not reported
Ou et al [32]	Three VR exercise games: Ocean Manager, Fruit Train, and Fishing Master.	HTC VIVE	Attention, cognitive ability, abstract reasoning, and complex information processing	36 sessions over 12 weeks; 40 min per session (30 min of game play and 10 min of breaks)
Rodrigo-Yanguas et al [53]	VR serious game, The Secret Trail of the Moon	—	Executive functions	12 sessions over 12 weeks; 40 min per session
Schena et al [54]	IAmHero VR system with 3 serious games: Topological Categories, Infinite Runner, and Space Coding.	Oculus Quest 2	ADHD symptoms and executive functions	24 treatment sessions over 6 months; 30 min per session
Selaskowski et al [31]	CPT with distractor elements in a virtual seminar room with gaze-based attention refocusing training	HTC Vive Pro Eye	Attention and inhibitory control	One session lasting 1 hour, comprising 3 × 18 min blocks (1 for each treatment) with 2 min breaks
Sergis et al [28]	ADHD Dog with 3 serious games: Cross the Road, Park Attention Shooter, Memory Market	Meta Quest 2 HMD	Attention, executive function, and memory	6 months of game play (frequency and duration of sessions not reported)
Skalski et al [26]	Participants observed flash game images on a computer screen located in a virtual room with or without distractors.	HTC VIVE Cosmos Elite	Executive attention	10 sessions over 10 weeks; 30 min per session
Tabrizi et al [33]	VR 360degree videos with target imagery and audio-visual intrusive stimuli.	VR Box	Memory	10 sessions; 3 min per session (frequency of sessions not reported)
Wong et al [23]	VR simulation of real-life social situations: classroom, mass transit railway station and train, a supermarket and restaurant	—	Social skills, self-control, initiative, and emotional control	12 sessions over 3 weeks; 20 min per session.

a VR: virtual reality.

b CPT: continuous performance test.

c Not available (not reported).

d ADHD: attention-deficit/hyperactivity disorder.

e ATA: advanced test of attention.

### Research Designs and Comparators

Participant VR use was appraised in 8 RCT studies, 5 quantitative nonrandomized studies, 1 quantitative descriptive study, and 1 mixed methods study (Table 1). Comparators included medication, psychotherapy, therapeutic chess, hemoencephalography, biofeedback with physical activity training, occupational therapy, speech and psychomotor treatment, social skills training, a healthy control group, and a no-treatment control group. All included studies reported pretest and posttest measurements on user outcomes. One study also reported a 2-month follow-up measurement [33].

### Effectiveness Outcomes

Table 3 presents the study measures and outcomes of cognitive rehabilitation with immersive VR for people with ADHD. Effect sizes from the included studies that reported them, or reported sufficient data to permit their calculation, are summarized in Table 4 (always reported as Hedges *g* to facilitate comparisons). Findings generally indicate that VR-based interventions either improve cognitive performance for children and adults with ADHD or have no significant impact. In Table 4, positive effect sizes denote an improvement from pretest to posttest (ie, better performance, fewer errors, reduced symptoms, etc) or a favorable comparison between the VR group and its comparator. Regarding pre-post comparisons, effect sizes were overwhelmingly in favor of VR having a positive impact on omission and commission errors (−0.089 to 2.283, with 8 of 9 effects being positive [26,35,51]), attention (0.360 to 3.321 [35,54]), executive functioning (−0.159 to 2.775, with 13 of 14 effects being positive [23,26,30,35,49,54]), processing speed (1.250 to 1.527 [25,26]), working memory (0.160‐1.520 [25,33,54]) and impulsivity (0.260 to 2.420 [23,35,54]). Between-groups comparisons of posttest measures returned uniformly positive effect sizes for omission and commission errors (0.462‐1.774 [26,35,51]), executive function (0.235 to 1.037 [23,26]), and processing speed (0.678 to 1.031 [25,26]). Effect size estimates for attention (−2.092 to 0.677 [28,35,50]), working memory (−0.705 to 3.278 [25,33]), and impulsivity (−1.361 to 0.467 [23,35,50]) fell either side of zero.

**Table 3.** Effectiveness outcomes of cognitive training with immersive virtual reality (VR) for attention-deficit/hyperactivity disorder (ADHD).

Study and measures	Effectiveness outcomes
Bioulac et al [35]	
ADHD Rating Scale IV—Parent Version	No significant differences reported for the VR group from before to after intervention.
Continuous Performance Test II (postintervention)	Significantly fewer commission errors in the VR group compared with the methylphenidate group (*P*<.05). No differences in omissions between VR and methylphenidate groups.
Virtual Classroom Assessment Test (postintervention)	Significantly more correct hits in the VR group compared with the psychotherapy group (*P*<.0001). No difference in correct hits between the VR and methylphenidate groups (*P*>.05). Significantly fewer commission errors in the VR group compared with the methylphenidate group (*P*<.0001).
Cuber et al [49]	
7-item Concentration Questionnaire; items derived from Adult Concentration Inventory	Significant decrease (improvement) in concentration score between pretest and first session (P<.0001), and between pretest and mean across all sessions (*P*<.0001). Concentration score did not change across sessions (*P*>.92)
Cunha et al [25]	
The Southwestern Assessment of Processing Speed (SWAPS)	Significant increase in processing speed from preintervention to postintervention for the VR group (*P*<.001). No change in processing speed from preintervention to postintervention for the control group (*P*=.68). No significant difference in processing speed between the VR and control groups postintervention (*P*=.104).
Sequence of Letters and Numbers and Spatial Location subtests from the Wechsler Adult Intelligence Scale—Third Edition (WAIS-III)	Significant increase in working memory (spatial location) from preintervention to postintervention for the VR group (*P*=.034) and no change for the control group (*P*=.167), but no significant difference between VR and control groups postintervention (*P*=.183). No significant increase in working memory (sequence of letters and numbers) from preintervention to postintervention for the VR group (*P*=.465) and no significant difference between the VR and control groups postintervention (*P*=.052).
David et al [50]	
ADHD-rating scale IV—Home Version (Romanian)	Significantly fewer parent-reported overall ADHD symptoms after treatment in the VR combined with medication group compared with the medication-only group. (Cohen *d*=1.30, *P*=.002). No significant differences in parent-reported overall ADHD symptoms in the combined VR-medication group compared with VR therapy alone group. (Cohen *d*=0.04, *P*=.916)
ADHD-rating scale IV—School Version (Romanian)	No significant differences in teacher-reported overall ADHD symptoms between the VR-medication group compared with the medication-only (Cohen *d*=0.57, *P*=.259) or VR therapy-only (Cohen *d*=0.04, *P*=.916) groups.
The Child Behavior Checklist (teacher- and parent-reported comorbid internalizing and externalizing problems)	No significant differences in parent-reported internalizing or externalizing problems between the VR-medication group compared with the medication-only (Cohen *d*=−0.21, *P*=.577) or VR therapy-only (Cohen *d*=−0.46, *P*=.226) groups. No significant differences in teacher-reported internalizing / externalizing problems between the VR combined group compared with the medication-only (Cohen *d*=−0.32/0.56, *P*=.198/.072) or VR therapy-only (Cohen *d*=−0.35/‐0.07, *P*=.177/.426) groups.
De Luca et al [30]	
Italian Battery for the Assessment of Children with ADHD (BIA) for executive functions—Frog Test of attentional and control processes	Significant improvement in attentional processes from preintervention to postintervention for children in the BRAVO games experimental group and not the traditional therapy control group (*P* time x group interaction=.03)
Battery for assessing language in children 4-12 years (BVL)	Children from both groups (BRAVO games and traditional therapy) demonstrated improved language performance from preassessment to postassessment on BVL subtests of phonological fluency, semantic fluency, repetition of phrases, and repetition of nonwords (*P* time=.01) Significant improvement in BVL grammatical comprehension from preintervention to postintervention for children in the BRAVO games experimental group and not the control group (*P* time × group interaction=.02)
OMINO GOODENOUGH	Children from both groups (BRAVO games and traditional therapy) demonstrated improved self-perception, body representation, and emotional expression as assessed by the OMINO GOODENOUGH from preassessment to postassessment (*P* time=.01)
Visual Motor Integration (VMO)	Children from both groups (BRAVO games and traditional therapy) demonstrated improved visual-motor integration skills from preassessment to postassessment (*P* time=.04)
Topographical Categories Game—topographical concepts, ability to wait and follow rules	Significantly improved performance on the Topographical Categories game over time from preassessment to the final assessment at 6 months (*P*<.0001) despite increasing difficulty in game levels.
Infinite Runner Game—concentration, motor coordination, ability to wait and follow rules	Significantly improved performance on the Infinite Runner game over time, with the largest difference occurring between preassessment and the fifth month intervention assessment (*P*<.001) despite increasing difficulty in game levels.
Planning Game—planning, problem-solving, and social interaction skills	Significantly improved performance on the Planning game over time, with the largest difference occurring from the third month of assessment to the final assessment when performance peaked (*P*<.001), despite increasing difficulty in game levels.
Kim et al [51]	
Advanced Test of Attention (ATA), Errors of Omission	Significant reduction in omission errors (*P*<.001), in the experimental group from preintervention to postintervention. Fewer postintervention omission errors, in the experimental group versus the control group (*P*<.001).
Advanced Test of Attention (ATA), Errors of Commission	Significant reduction in commission errors (*P*<.01), in the experimental group from preintervention to postintervention. Fewer postintervention omission errors, in the experimental group versus the control group (*P*<.001).
Oh et al [52]	
Electroencephalography	No significant changes preintervention to postintervention in alpha, beta, delta, or gamma brainwave patterns were found after VR cognitive training (*P* values >.10). No significant differences posttest comparing experimental group with control group in alpha, beta, delta, or gamma brainwave patterns (*P* values >.09).
Ou et al [32]	
Test of Nonverbal Intelligence (Fourth edition)	Regarding the performance in intelligence, cognitive function, and critical thinking ability, one participant increased their score from 78 to 87, one retained a score of 84, and one increased their score from 65 to 83.
Attention Test for Elementary School Children	Scores improved on focused, sustained, selective, alternating, and divided attention.
Wisconsin Card Sorting Test	Posttest, one participant improved in abstract reasoning abilities, information processing abilities, and sustained responses, while the others showed reduced performance. One participant had fewer perseverative errors and nonperseverative errors, while 2 did not improve their performance and showed increased perseverative errors and nonperseverative errors.
Swanson, Nolan, and Pelham Questionnaire—Parent Version (Chinese)	Parents reported improvements in attention, hyperactivity/impulsivity, and behaviors of oppositional defiant disorder after the experiment.
Rodrigo-Yanguas et al [53]	
Behavior Rating Inventory of Executive Function-2 (BRIEF-2)	There were no significant improvements in BRIEF-2 executive functioning posttest for the VR experimental intervention, The Secret Trail to the Moon (TSTM) (*P* values >.10).
ATENTO Questionnaire	The per protocol analysis showed no significant differences in ATENTO parent report scores between the VR experimental TSTM, Therapeutic Chess (TC), and control groups (*P* values >.17). Significant difference between the TSTM and control group on the ATENTO self-report scores of school context, favoring TSTM (*P*=.03). No other differences between the TSTM and control group on ATENTO self-report scores (*P* values >.17).
Quantitative Scales by Swanson, Nolan, and Pelham (SNAP-IV)	No significant differences in SNAP-IV scores between the VR experimental TSTM group, TC group, and control group (*P* values >.24).
Conners Parent Rating Scale (CPRS-HI)	No significant differences in CPRS-HI scores between the VR experimental TSTM group, TC group, and control group (*P*=.64).
Conners Continuous Performance Test 3 (CPT-3)	Significant improvement in block change for the VR TSTM group compared with the control group (*P*=.04). Significant improvement in interstimulus change for the VR TSTM group compared with the TC group (*P*=.01) and control group (*P*=.01).
Schena et al [54]	
Italian Battery for ADHD	Significant improvements preintervention to postintervention in sustained visual attention (*P*=.001), impulsive behavior (*P*=.045), and selective auditory attention (*P*=.001). No significant differences were reported for inhibitory control (*P*=.513), or motor inhibition (*P*=.853).
Tower of London	Significant improvements preintervention to postintervention in planning and organization (*P*=.013), problem-solving (*P*=.004), and executive functions (*P*=.035).
Conners-3	Significant improvements preintervention to postintervention in scores on hyperactivity/impulsivity (*P*=.03), learning-related problems (*P*=.01), and provocation/aggressiveness (*P*<.001). However, no significant improvement was found for inattention (*P*=.208).
Selaskowski et al [31]	
CPT Performance	No differential effect of feedback on the ADHD group compared with the control group for either omission errors (*P* feedback×group=.293), commission errors (*P* feedback×group=.372), or reaction time (*P* feedback×group=.982).
Sergis et al [28]	
Likert Scale Feedback Questionnaire for Participants with ADHD to assess Cognitive Functions and User Experience	Participants playing ADHD Dog displayed significantly higher self-reported ratings of focus and regulated attention (*P*=.002), self-efficacy in managing ADHD symptoms (*P*<.001), and an overall positive influence on their daily activities (*P*<.001) compared with active controls.
Skalski et al [26]	
Vigilance—Mackworth Clock Task (Short Form)	No differences between groups on commission or omission errors at pretest (*P* values >.50). Pretest to posttest improvements observed in omission and commission errors in the biofeedback+VR groups with and without distractors. At posttest, fewer omission and commission errors were observed for the biofeedback+VR groups with (*P* values *<*.004) and without (*P*<.002) distractors compared with the biofeedback-only group.
Visual Search Task (RT slope)	No differences between groups on reaction time slope at pretest (*P*=.391). Pretest to posttest improvements observed in reaction time slope in the biofeedback+VR groups with and without distractors. At posttest, a shallower (more efficient) reaction time slope was observed in biofeedback+VR groups with (*P*=.004) and without (*P*<.001) distractors, compared with the feedback-only group.
Multitasking Test (RT)	No differences between groups on single- or multi-task reaction times at pretest (*P* values >.30). Pretest to posttest improvements observed in single- and multi-task reaction times in the biofeedback+VR groups with and without distractors. At posttest, faster single- and multi-task reaction times were observed in the biofeedback+VR groups with (*P* values <.009) and without (*P* values <.003) distractors, compared with the feedback-only group.
Tabrizi et al [33]	
Digit Span Subscale of Wechsler’s Intelligence Scale for Children (working memory)	No differences between groups on pretest working memory performance (*P* values >.05). Improvement in pretest to posttest working memory scores for the VR group (Hedges *g*=1.520). At posttest, VR groups exhibited significantly better working memory performance than the control (*P*=.001) or medicine (*P*=.001) groups.
Wong et al [23]	
Social Skills Improvement System Rating Scale—Parents	No differences between the 3 groups on any measure at pretest (*P* values >.17). The VR training group achieved improved scores from pretest to posttest for self-control (Cohen *d*=0.46), initiative (Cohen *d*=0.16), and cooperation (Cohen *d*=0.06). At posttest, the VR training group achieved significantly higher self-control ratings than the traditional training (*P*=.047, Cohen *d*=0.44) and the waitlist control (*P*=.047, Cohen *d*=0.44) groups. There were no posttest significant differences in parent ratings of initiative between the VR training group and the traditional training (*P*=.180, Cohen *d*=0.24) or waitlist control (*P*=.065, Cohen *d*=0.40) groups. There were no posttest significant differences in parent ratings of cooperation between the VR training group and the traditional training (*P*=.053, Cohen *d*=0.42) or waitlist control (*P*=.398, Cohen *d*=0.07) groups.
Modified Riggio Basic Social Skills Assessment—Clinical Psychologist	No differences between the 3 groups on clinician ratings at pretest (*P*=.49). The VR training group achieved improved clinician ratings from pretest to posttest (Cohen *d*=2.42). The VR training group achieved significantly higher clinician ratings than the traditional training (*P*<.001, Cohen *d*=1.50) and waitlist control (*P*<.001, Cohen *d*=3.29) groups.
Behavior Rating Inventory of Executive Function—Parents	The VR training group demonstrated improved emotional control (Cohen *d*=0.46), but slightly poorer inhibition (Cohen *d*=−0.16) from pretest to posttest. At posttest, the VR training group did not differ significantly better from the traditional training group on emotional control (*P*=.064, Cohen *d*=0.40) or on inhibition (*P*=.183, Cohen *d*=0.24). At posttest, the VR training group exhibited superior emotional control (*P*=.005, Cohen *d*=0.68) and inhibition (*P*=.024, Cohen *d*=0.52) compared with the waitlist control group.

**Table 4.** Effect sizes from cognitive training with immersive VR^a^ for ADHD^b^.

Design and cognitive skill	Study	Comparison groups	Hedges *g*
Within group (prepost)			
Omission errors			
Continuous Performance Test	Bioulac et al [35]	Virtual classroom cognitive remediation group (pre vs post)	−0.089
Advanced Test of Attention	Kim et al [51]	Group experiencing VR training with pulmonary biofeedback (pre vs post)	1.247
Mackworth Clock Task	Skalski et al [26]	Group experiencing VR+biofeedback, without distractors (pre vs post)	1.826
Mackworth Clock Task	Skalski et al [26]	Group experiencing VR+biofeedback, with distractors (pre vs post)	1.568
Commission errors			
Continuous Performance Test	Bioulac et al [35]	Virtual classroom cognitive remediation group (pre vs post)	0.480
Virtual classroom commission errors	Bioulac et al [35]	Virtual classroom cognitive remediation group (pre vs post)	0.907
Advanced Test of Attention	Kim et al [51]	Group experiencing VR training with pulmonary biofeedback (pre vs post)	0.442
Mackworth Clock Task	Skalski et al [26]	Group experiencing VR+biofeedback, without distractors (pre vs post)	2.283
Mackworth Clock Task	Skalski et al [26]	Group experiencing VR+biofeedback, with distractors (pre vs post)	2.278
Attention			
ADHD-RS^c^ (inattention)	Bioulac et al [35]	Virtual classroom cognitive remediation group (pre vs post)	0.663
Italian Battery ADHD (sustained visual attention)	Schena et al [54]	Group exposed to VR serious games (pre vs post)	0.767
Italian Battery ADHD (auditory attention)	Schena et al [54]	Group exposed to VR serious games (pre vs post)	3.321
Conners-3 (inattention)	Schena et al [54]	Group exposed to VR serious games (pre vs post)	0.360
Executive function			
Adult Concentration Inventory	Cuber et al [49]	VR experience group (pre vs post)	2.775
Topographical Categories Game	De Luca et al [30]	Group exposed to VR serious games (pre vs post)	2.439
Infinite Runner Game	De Luca et al [30]	Group exposed to VR serious games (pre vs post)	1.221
Planning Game	De Luca et al [30]	Group exposed to VR serious games (pre vs post)	1.221
Tower of London (planning)	Schena et al [54]	Group exposed to VR serious games (pre vs post)	1.443
Tower of London (problem-solving)	Schena et al [54]	Group exposed to VR serious games (pre vs post)	2.745
Tower of London (executive function)	Schena et al [54]	Group exposed to VR serious games (pre vs post)	0.684
Italian Battery ADHD (inhibitory control)	Schena et al [54]	Group exposed to VR serious games (pre vs post)	1.143
Italian Battery ADHD (motor inhibition)	Schena et al [54]	Group exposed to VR serious games (pre vs post)	0.088
Multitasking Test (multitask reaction time)	Skalski et al [26]	Group experiencing VR+biofeedback, without distractors (pre vs post)	1.776
Multitasking Test (multitask reaction time)	Skalski et al [26]	Group experiencing VR+biofeedback, with distractors (pre vs post)	1.198
Initiative (parent rating)	Wong et al [23]	VR training group (pre vs post)	0.157
BRIEF-parent^d^ (emotional control)	Wong et al [23]	VR training group (pre vs post)	0.457
BRIEF-parent (inhibition)	Wong et al [23]	VR training group (pre vs post)	−0.159
** **Processing speed			
SWAPS^e^	Cunha et al [25]	VR group (pre vs post)	1.250
Visual Search Task (reaction time slope)	Skalski et al [26]	Group experiencing VR+biofeedback, without distractors (pre vs post)	1.527
Visual Search Task (reaction time slope)	Skalski et al [26]	Group experiencing VR+biofeedback, with distractors (pre vs post)	1.269
Multitasking Test (single task reaction time)	Skalski et al [26]	Group experiencing VR+biofeedback, without distractors (pre vs post)	1.477
Multitasking Test (single task reaction time)	Skalski et al [26]	Group experiencing VR+biofeedback, with distractors (pre vs post)	1.496
Working memory			
Spatial location	Cunha et al [25]	VR group (pre vs post)	0.581
Letters/numbers sequence	Cunha et al [25]	VR group (pre vs post)	0.160
Conners-3 (learning-related problems)	Schena et al [54]	Group exposed to VR serious games (pre vs post)	0.643
WIS^f^ for children (Digit Span Subscale)	Tabrizi et al [33]	VR therapy group (pre vs post)	1.520
Hyperactivity and impulsivity			
ADHD-RS (hyperactivity)	Bioulac et al [35]	Virtual classroom cognitive remediation group (pre vs post)	0.260
Italian Battery ADHD (impulsivity)	Schena et al [54]	Group exposed to VR serious games (pre vs post)	2.420
Conners-3 (hyperactivity/impulsivity)	Schena et al [54]	Group exposed to VR serious games (pre vs post)	0.874
Conners-3 (provocation/aggression)	Schena et al [54]	Group exposed to VR serious games (pre vs post)	0.475
Self-control (parent rating)	Wong et al [23]	VR training group (pre vs post)	0.453
Miscellaneous			
Virtual classroom direct hits	Bioulac et al [35]	Virtual classroom cognitive remediation group (pre vs post)	1.209
Cooperation (parent rating)	Wong et al [23]	VR training group (pre vs post)	0.058
Clinician-assessed social skills	Wong et al [23]	VR training group (pre vs post)	2.389
Between groups (posttest)			
Omission errors			
Continuous Performance Test	Bioulac et al [35]	Virtual classroom cognitive remediation group versus psychotherapy placebo group	1.402
Continuous Performance Test	Bioulac et al [35]	Virtual classroom cognitive remediation group versus psycho stimulant group	0.568
Advanced Test of Attention	Kim et al [51]	Group experiencing VR training with pulmonary biofeedback versus active control group	1.774
Mackworth Clock Task	Skalski et al [26]	Group experiencing VR+biofeedback vs biofeedback only, without distractors	0.982
Mackworth Clock Task	Skalski et al [26]	Group experiencing VR+biofeedback vs biofeedback only, with distractors	0.753
Commission errors			
Continuous Performance Test	Bioulac et al [35]	Virtual classroom cognitive remediation group versus psychotherapy placebo group	0.462
Continuous Performance Test	Bioulac et al [35]	Virtual classroom cognitive remediation group versus psycho stimulant group	0.798
Virtual Classroom	Bioulac et al [35]	Virtual classroom cognitive remediation group versus psychotherapy placebo group	0.519
Virtual Classroom	Bioulac et al [35]	Virtual classroom cognitive remediation group versus psycho stimulant group	0.808
Advanced Test of Attention	Kim et al [51]	Group experiencing VR training with pulmonary biofeedback versus active control group	0.773
Mackworth Clock Task	Skalski et al [26]	Group experiencing VR+biofeedback vs biofeedback only, without distractors	0.807
Mackworth Clock Task	Skalski et al [26]	Group experiencing VR+biofeedback vs biofeedback only, with distractors	0.757
Attention			
ADHD-RS (inattention)	Bioulac et al [35]	Virtual classroom cognitive remediation group versus psychotherapy placebo group	−0.542
ADHD-RS (inattention)	Bioulac et al [35]	Virtual classroom cognitive remediation group versus psycho stimulant group	−2.092
ADHD-RS (parent) (inattention)	David et al [50]	VR therapy+medication group versus medication only group	0.418
ADHD-RS (teacher) (inattention)	David et al [50]	VR therapy+medication group versus medication only group	0.326
Focus and regulated attention	Sergis et al [28]	VR group playing (serious game) ADHD Dog versus active control group	0.677
Executive function			
Multitasking Test (multitask reaction time)	Skalski et al [26]	Group experiencing VR+biofeedback vs biofeedback only, without distractors	1.037
Multitasking Test (multitask reaction time)	Skalski et al [26]	Group experiencing VR+biofeedback vs biofeedback only, with distractors	0.647
Initiative (parent rating)	Wong et al [23]	VR training group versus traditional training group	0.235
Initiative (parent rating)	Wong et al [23]	VR training group versus waitlist control	0.391
BRIEF-parent (emotional control)	Wong et al [23]	VR training group versus traditional training group	0.393
BRIEF-parent (emotional control)	Wong et al [23]	VR training group versus waitlist control	0.675
BRIEF-parent (inhibition)	Wong et al [23]	VR training group versus traditional training group	0.232
BRIEF-parent (inhibition)	Wong et al [23]	VR training group versus waitlist control	0.513
Processing speed			
SWAPS	Cunha et al [25]	VR group versus control group	0.678
Visual Search Task (reaction time slope)	Skalski et al [26]	Group experiencing VR+biofeedback vs biofeedback only, without distractors	1.031
Visual Search Task (reaction time slope)	Skalski et al [26]	Group experiencing VR+biofeedback vs biofeedback only, with distractors	0.729
Multitasking Test (single task reaction time)	Skalski et al [26]	Group experiencing VR+biofeedback vs biofeedback only, without distractors	0.789
Multitasking Test (single task reaction time)	Skalski et al [26]	Group experiencing VR+biofeedback vs biofeedback only, with distractors	0.772
Working memory			
Spatial location	Cunha et al [25]	VR group versus control group	−0.551
Letters/numbers sequence	Cunha et al [25]	VR group versus control group	−0.705
WIS for children (Digit Span Subscale)	Tabrizi et al [33]	VR therapy group versus control group	3.278
WIS for children (Digit Span Subscale)	Tabrizi et al [33]	VR therapy group versus medicine group	1.244
Hyperactivity and impulsivity			
ADHD-RS (hyperactivity)	Bioulac et al [35]	Virtual classroom cognitive remediation group versus psychotherapy placebo group	−0.474
ADHD-RS (hyperactivity)	Bioulac et al [35]	Virtual classroom cognitive remediation group versus psycho stimulant group	−1.361
ADHD-RS (parent) (hyperactivity)	David et al [50]	VR+medication group versus medication only group	0.467
ADHD-RS (teacher) (hyperactivity)	David et al [50]	VR+medication group versus medication only group	0.219
Self-control (parent rating)	Wong et al [23]	VR training group versus traditional training group	0.433
Self-control (parent rating)	Wong et al [23]	VR training group versus waitlist control	0.433
Internalizing and externalizing symptoms			
CBC^g^—Externalizing Problems (parent)	David et al [50]	VR+medication group versus medication only group	−0.156
CBC—Externalizing Problems (teacher)	David et al [50]	VR+medication group versus medication only group	0.545
CBC—Internalizing Problems (parent)	David et al [50]	VR+medication group versus medication only group	−0.205
CBC—Internalizing Problems (teacher)	David et al [50]	VR+medication group versus medication only group	0.312
Miscellaneous			
Virtual classroom correct hits	Bioulac et al [35]	Virtual classroom cognitive remediation group versus psychotherapy placebo group	1.401
Virtual classroom correct hits	Bioulac et al [35]	Virtual classroom cognitive remediation group versus psycho stimulant group	−.281
Self-efficacy in managing ADHD	Sergis et al [28]	VR group playing (serious game) ADHD Dog versus active control group	1.620
Positive influence of game on daily activities	Sergis et al [28]	VR group playing (serious game) ADHD Dog versus active control group	1.226
Cooperation (parent rating)	Wong et al [23]	VR training group versus traditional training group	0.417
Cooperation (parent rating)	Wong et al [23]	VR training group versus waitlist control	0.066
Clinician-assessed social skills	Wong et al [23]	VR training group versus traditional training group	1.478
Clinician-assessed social skills	Wong et al [23]	VR training group versus waitlist control	3.246

a VR: virtual reality.

b ADHD: attention-deficit/hyperactivity disorder.

c ADHD-RS: attention-deficit/hyperactivity disorder—rating scale.

d BRIEF-parent: behavior rating inventory of executive function (parent rated).

e SWAPS: The Southwestern Assessment of Processing Speed.

f WIS: Wechsler Intelligence Scale (for children).

g CBC: Child Behavior Checklist.

A total of 10 studies evaluated standalone VR [25,28,30,31,33,35,49,52-54], 4 evaluated adjunctive VR [23,26,50,51], and 1 examined both approaches [32]. Treatment effect sizes exhibited comparable magnitudes across both standalone [25,28,30,33,35,49,54] and adjunctive VR interventions [23,26,50,51]. While most studies used significance levels of *P*<.05, one study set its significance level at *P*=.10 [53]. Interventions that included VR outperformed traditional social skills groups [23], stimulant and nonstimulant medications [35,50], a psychomotor program [51], 2D hemoencephalography biofeedback [26], therapeutic chess, and passive control groups [53] in addressing various ADHD symptoms.

When attention is considered more broadly through self-reports, other informants, and performance-based measures, standalone VR currently has a larger body of evidence supporting improvements [28,30,49,53,54]. However, for specific performance metrics such as omission and commission errors on the continuous performance test (CPT), adjunctive VR again shows stronger effects, with 2 studies reporting consistently significant reductions [26,51]. Evidence from standalone VR on the CPT is limited to a single study, which found significant improvements in commission errors but not omissions compared with a control group [35].

Beyond attention, some standalone VR studies demonstrate improvements in hyperactivity, impulsivity [54], and inhibitory control [30,53], though others show no effect on inhibitory control [31,54]. Standalone VR has demonstrated effectiveness in improving functioning across various cognitive and executive domains, such as planning, memory, and problem-solving [30,33,53,54]. In contrast, findings for adjunctive VR are mixed; one study reported moderate improvements in emotional control [23], while another found inconsistent outcomes for reasoning and information processing [32]. One adjunctive VR study reported a small, significant deterioration in inhibition based on parent ratings from pretest to posttest [23]. Notably, standalone VR produced a large effect on memory in one study and outperformed medication [33].

### User Experience With the VR Interventions

Table 5 shows that attrition rates during VR-based treatment (mean 7.0%, SD 8.1%, range 0%‐41%) were lower than overall study attrition rates (mean 11.7%, SD 12.2%, range 0%‐41%). Three studies used an intention-to-treat analysis [23,50,53]. Most studies used nonstandardized measures (ie, questions and observations) of VR user experience factors such as immersion, acceptability, adverse effects, cybersickness, and engagement. Two studies used a standardized measure of VR user presence [51,52], and one study [30] relied on biometric indicators. There were limited cases of cybersickness reported, including motion sickness [49], headaches [49], nausea [50], dizziness [50], and eye strain [49], as well as discomfort due to the headset [25,49]. Three studies reported an absence of cybersickness symptoms and other adverse events [31,33,35].

**Table 5.** Findings on VR^a^ user engagement outcomes.

Study	Attrition (%)	Engagement measures	Engagement outcomes
Bioulac et al [35]	VR group: 3/19 (16); Methylphenidate group: 4/20 (20); Psychotherapy group: 2/21 (10)	Nonstandardized measure	No cybersickness-related side effects reported.
Cuber et al [49]	11/27 (41)	System usability scale and qualitative feedback	Eight participants reported discomfort associated with the weight of the headset and eye strain. Four participants experienced headaches. One participant reported motion sickness and withdrew from the study. Most participants adjusted within 1 to 6 sessions of VR use. System usability was rated as good (mean 72.71).
Cunha et al [25]	VR: 0/13 (0); Passive control group: 1/13 (1)	Nonstandardized satisfaction questionnaire	Four participants reported difficulty with use of the helmet and game controls.
David et al [50]	VR with psychotherapy and atomoxetine: 5/20 (25); Atomoxetine: 7/21 (33); VR with psychotherapy: 4/18 (22)	Nonstandardized measure, vital signs monitoring	Participants in the VR groups reported mild side effects, including appetite decrease (n=5), abdominal pain (n=2), nausea (n=1), pruritus (n=1), somnolence (n=2), dizziness (n=1), irritability (n=5), and increased systolic blood pressure (n=1). No participants reported severe adverse events.
De Luca et al [30]	VR group: 0/30 (0); Traditional therapy group: 1/30 (3)	Biofeedback parameters (galvanic skin response, temperature, blood volume pulse, and interbeat interval)	Excitement indicated by galvanic skin responses correlated positively with performance in some games, but not in others. However, when a child did very well, galvanic skin responses dropped back to average levels, possibly indicating calm focus and mastery rather than stress.
Kim et al [51]	Nil	Slater-Usoh-Steed Presence Questionnaire and qualitative feedback	Qualitative responses suggested participants experienced high levels of immersion, presence, enjoyment, and ease of use.
Oh et al [52]	Nil	Sense of presence questionnaire	The sense of presence after VR cognitive training significantly improved in the ADHD^b^ group (*P*=.02) but not the control group.
Ou et al [32]	Nil	Nonstandardized measure	Participants were observed to lose enthusiasm after mastering each game. However, enthusiasm returned when they could choose which games to play in the sessions. Participants had a 5-minute break after each 10 min game to prevent nausea and headset discomfort.
Rodrigo-Yanguas et al [53]	VR serious game: 4/35 (11); Therapeutic chess: 10/34 (29); Control intervention: 1/35 (3)	Not measured	Not reported
Schena et al [54]	VR: 0/30 (0); Speech and psychomotor therapy: not reported	Nonstandardized measure	The VR approach was deemed acceptable and flexible by the authors.
Selaskowski et al [31]	2/38 (5); Not reported separately for each group	VRSQ (Virtual Reality Sickness Questionnaire)	No interruptions caused by discomfort and no participants reported any cybersickness or other adverse events
Sergis et al [28]	Nil	Nonstandardized measure	Participants reported high levels of comfort and ease of use
Skalski et al [26]	BF^c^: 0/30 (0); VR-BFD^d^: 2/30 (7); VR-BF^e^: 1/30 (3)	None reported	None reported
Tabrizi et al [33]	Nil	Nonstandardized measure	High immersion and no negative side effects reported
Wong et al [23]	VR-based social skills training: 0/30 (0); Conventional social skills training: 1/30 (3); Waitlist control: 11/30 (37)	Simulator sickness questionnaire and nonstandardized measure of satisfaction	High acceptance and satisfaction with the VR training reported by participants; no participants reported discomfort or other cybersickness symptoms

a VR: virtual reality.

b ADHD: attention-deficit/hyperactivity disorder.

c BF: standard hemoencephalographic biofeedback with physical activity training.

d VR-BFD: virtual reality and hemoencephalographic biofeedback with distractors.

e VR-BF: virtual reality and hemoencephalographic biofeedback without distractors.

### Quality Assessment Results

All RCTs reported the use of randomization to assign participants to treatment groups [23,25,26,33,35,50,53,54]; however, the randomization method was only specified in 4 RCTs [23,26,50,53]. In all the RCT studies, participants reportedly adhered to their assigned VR intervention. With the exception of 4 studies [23,35,53,54], the remaining RCT studies reported comparable baseline group analyses and complete outcome data defined as >80% [25,26,33,50]. Two studies had blinding of outcome assessors, which was applied at pretest measurements [23,50]. In the quantitative nonrandomized studies [28,30,31,51,52], participants were recruited in a way that minimized selection bias, measures were appropriate, the intervention was administered as intended, and there was complete outcome data in most cases [28,31,51,54]. Participant groups were not comparable in one study, as the control group did not consist of ADHD participants [52]. In the quantitative descriptive study [32], the sampling strategy was relevant to address the research question, the sample was representative of the target population, measurements and statistical analyses were appropriate, and the risk of nonresponse bias was low. In the mixed methods study [49], questionnaires were administered before and after VR sessions, and data were analyzed using *t* tests, which was an appropriate statistical method. A thematic analysis was performed on interview data, which was appropriate to answer the research questions. Quantitative and qualitative findings were well integrated, and any inconsistencies were addressed.

## Discussion

### Principal Findings

#### Overview

This systematic review sought to comprehensively identify studies that examined the use of immersive VR technology for people with ADHD, report on available treatment and user experience evidence regarding cognitive rehabilitation, and also identify research gaps for future exploration in this domain. A total of 15 eligible studies were found in this review. The reported effectiveness outcomes from immersive VR-based interventions were generally positive, with improvements in ADHD cognitive symptoms. It was also found that immersive VR technology is well-received with positive user experiences reported by participants and a low attrition rate observed across all studies. However, the low number of relevant studies available reveals key treatment and research gaps for future studies to address.

#### Effectiveness Outcomes

Immersive VR-based interventions procured significant improvements in cognitive abilities for children with ADHD [26,30,32,33,35,51,54]. Consistent improvements were observed for omission and commission errors, executive functions (including inhibitory control, planning, organization, and problem-solving), and processing speed. Attention, working memory, and impulsivity also benefited across most comparisons. In 5 studies, VR-based interventions outperformed active controls, including medication [35,50] and standard therapies [26,30,35,51]. Treatment effect sizes were most often medium to large across studies, which is congruent with previous research on the observed magnitude of VR treatment effectiveness [8,55,56]. There were a few instances in which postintervention effects were marginally in favor of non-VR treatment groups compared with VR-based interventions with respect to cognitive performance or ADHD symptoms [25,35,50]. However, these only constituted a minimal proportion of the total observations reviewed. In general, VR-based interventions constitute a benign-to-beneficial modality for ADHD cognitive rehabilitation. Furthermore, positive effectiveness outcomes were obtained from treatment sessions that ranged from 3 to 50 minutes. This supports the integration of immersive VR interventions into standard clinician treatment sessions, which typically have a time length of 50 to 60 minutes [57].

Adults who received VR interventions also showed significant improvements in attention or concentration after treatment [49] and compared with an active control [28]. However, the control incorporated elements of VR, so further research is needed to compare VR to independent conditions such as medication or psychotherapy in adults. One study found that processing speed and visual-spatial working memory improved post-VR, but no significant differences were found compared with passive controls [25]. Research in adults has focused on reducing distractions and increasing personal drive to perform productive behaviors. VR interventions significantly improve work efficiency and motivation [49] as well as self-efficacy and sense of achievement [28]. However, the impact of providing automated performance feedback during VR remains inconclusive. While studies found no statistical effect of performance feedback, some participants reported that they found feedback frustrating and disruptive, while others believed it helped them regulate and refocus [31,49].

One-third of the reviewed studies (n=5) had predominantly female participant samples. Of these, 3 investigated standalone VR interventions [25,49,52], one examined adjunctive VR [51], and one included both approaches [32]. The remaining 10 studies, which had predominantly male samples, included 7 standalone VR interventions [28,30,31,33,35,53,54] and 3 adjunctive ones [23,26,50]. Most of the female-dominant studies focused on attention (3/5, 60%), all of which reported significant improvements in concentration or reductions in omission and commission errors [32,49,51]. Similarly, most male-dominant studies (8/10, 80%) investigated attention, with the majority reporting significant improvements [26,28,30,35,50,53,54]. However, in some cases, outcomes varied depending on the measure used [35,50,53].

Notably, only one female-dominant study examined hyperactivity-impulsivity symptoms, and none assessed inhibitory control [32]. In contrast, most male-dominant studies (7/10, 70%) included at least one indicator of these symptoms. Among these, 2 participants reported significant improvements in inhibitory control [30,53], and one reported improvement in hyperactivity-impulsivity [53]. However, outcomes on informant-rated ADHD scales were mixed [35,50,53]. In addition to behavioral symptoms, few female-dominant studies investigated executive functions (2/5, 40%), and findings were inconsistent [25,32]. In contrast, a higher proportion of male-dominant studies assessed executive functions (5/10, 50%), with most reporting significant improvements in planning, problem-solving, organization, or working memory [30,33,54].

Collectively, these findings have important clinical implications for psychologists selecting VR-based interventions for children with ADHD. VR appears to be a promising tool for improving attention in both boys and girls. For boys, it also shows potential to enhance executive functioning, with some preliminary evidence suggesting it may also have a beneficial effect on hyperactivity-impulsivity symptoms. In contrast, evidence for these outcomes in girls is limited, indicating that clinicians should proceed with caution and consider supplementing VR with other interventions.

Caution is required in interpreting the effectiveness results. Some studies combined immersive VR with other treatments, such as medication, biofeedback, and occupational therapy [26,32,54], which limits attributions regarding the unique impact of the immersive VR component on cognitive abilities. Nevertheless, conditions containing a VR component reliably outperformed non-VR conditions, which highlights VR’s potential to supplement the treatments clinicians and clients are already using for better cognitive rehabilitation outcomes. Overall, combining VR with nonstimulant medication may be more effective than standalone VR for reducing parent-rated ADHD symptoms [35,50,53]. Standalone VR demonstrates consistent benefits across attention and executive functions, including planning, memory, inhibitory control, and problem-solving [30,33,53,54]. While adjunctive VR may be more effective in reducing specific attention errors [26,51], findings on its impact on executive functions remain limited [23,32].

Inconsistent results were found in some studies that assessed ADHD symptoms using multiple methods or informants (eg, CPT, parent, and teacher rating scales) [35,50,54]. For example, one study found that combined VR and medication outperformed medication alone for the treatment of ADHD based upon parent-rated symptoms but not teacher-rated symptoms [50]. As it stands, there are few studies that obtain teacher ratings for children in school or educator ratings for adults in tertiary education. Finally, no studies obtained follow-up measurements to determine the longevity of the cognitive improvements beyond the 2 months (reported by Tabrizi et al [33]). Therefore, it is unclear how long most of the observed immersive VR-based benefits will persist after treatment cessation.

#### User Experience

This review found immersive VR-based interventions to be generally safe for cognitive rehabilitation in children and adults with ADHD symptoms. These findings are consistent with past research showing little to no harmful effects from VR treatment [8,16,25]. There was also generally minimal attrition in the active VR treatment phase across the studies, which implies that VR-based treatments were motivating and engaging for the users. Even so, the small number of studies and the fact that most of them did not make use of standardized measures of user experience factors are limiting. Of those who offered commentary, Bioulac et al [35] reported that all participants completed their VR sessions with no adverse effects related to cybersickness. This suggests the technology is suitable for the virtual classroom, in line with previous research [34,58,59]. Nonetheless, several safety concerns were noted [25,49,50]. Some participants reported unpleasant reactions, including motion sickness, nausea, and dizziness, as well as headset discomfort. These adverse effects may have been at least jointly attributable to the participants’ concurrent use of ADHD medications, which can have side effects that mimic symptoms of cybersickness, including nausea, headaches, and increased blood pressure [60,61]. Conservatively, it is reasonably likely that a small minority of clients react negatively, at least initially, to the VR experience.

Several studies reported that VR was acceptable, comfortable, and easy to use [23,28,54] and achieved a high level of presence [52] or good immersion [33,51], which is consistent with previous research [8,62-64]. In one included study [32], enthusiasm diminished once the children mastered the assigned VR games. However, enthusiasm increased when they were given choices regarding which VR game to play next. These findings illuminate the need for clients, especially children, to have variety and choice in VR tasks to enhance enjoyment and effective outcomes [65]. User experience outcomes were not reported in the study, which integrated biofeedback into a VR environment [33]. Overall, evidence of adverse effects was scant, but clinicians should closely monitor VR use to foresee any adverse effects, particularly for those individuals who are also using pharmacotherapy with VR. For example, the study reporting the widest range of adverse effects found they were more prevalent in the combined VR and medication condition than in standalone VR, particularly appetite loss and irritability, suggesting that medication side effects may have contributed [50]. Furthermore, it is recommended that clinicians provide a choice and variety of personally relevant VR tasks to optimize user engagement and effectiveness, as research suggests these factors may increase user satisfaction, motivation, and reduce attrition [66].

All studies reporting no cybersickness symptoms or adverse effects included predominantly male participants [31,33,35]. Only one study reported on side effects in female participants [49], highlighting the need for further gender-specific research on user experience. The limited number of studies precludes the identification of consistent gender-based side effect profiles. Positive feedback regarding acceptability and immersion was reported across studies [23,28,33,51,52,54]. One female-dominant study noted reduced enthusiasm following game mastery [32], while a male-dominant study found lower galvanic skin response after mastery, suggesting reduced emotional arousal and a possible calming effect [30]. Clinicians should therefore consider whether game mastery leads to disengagement or a sense of calm and achievement, as individual responses may vary.

### Research Gaps and Future Research

This systematic review reveals several research gaps for further exploration. Included studies focused primarily on boys and children aged 5 to 15 years. As a result, there exists a research gap for girls, older adolescents, and adults of both sexes that could be explored. Moreover, the absence of specifying participants by ADHD presentation subtype (ie, predominantly inattentive, hyperactive-impulsive, or combined) and severity (as denoted in the *Diagnostic and Statistical Manual of Mental Disorders*, Fifth Edition, Text Revision) [1], precluded consideration of these factors. Furthermore, studies primarily originated from countries in Europe or Asia, which may limit the generalizability of findings to people with ADHD from other countries (eg, the United States, Canada, the United Kingdom, Australia, and New Zealand).

Consideration of the cost-effectiveness of immersive VR was beyond the scope of this review. However, some studies report lower costs [23,33,50,53] or greater feasibility [49] of VR interventions compared with conventional treatments. One study [50] suggested that moderate to large effect sizes would be needed to justify the cost of VR; thresholds that were met in several studies [23,25,26,33,49,50]. Despite these promising findings, further research into the cost-effectiveness of VR is warranted. Reusing equipment across participants may lower marginal costs, yet high initial expenses, including hardware, software licensing, and staff training, remain barriers to widespread adoption [23,67]. In particular, training clinicians or hiring VR-literate staff poses practical challenges, as such expertise is still uncommon. These challenges are amplified in low- and middle-income countries due to limited internet access, high data costs, and constrained institutional budgets [67]. Partnerships between universities, health care institutions, and technology companies may be critical to supporting sustainable VR implementation in clinical settings [67].

To improve methodological rigor, future research designs could include RCTs with increased sample sizes and follow-up measurements to appraise effect sizes and longer-term outcomes. Further comparisons of immersive VR interventions with standalone or combined psychotherapy and pharmacological treatments will confirm the value-adding potential of combined interventions and treatment. This research can help clarify which cognitive domains are most responsive to adjunctive VR and determine whether specific combinations of interventions yield more consistent benefits. To consolidate user experience findings and facilitate comparisons, the technology acceptance model is recommended [68]. Future research should adopt standardized user experience measures with known psychometric properties, such as the system usability scale [69] and the Simulator Sickness Questionnaire [70]. It is also recommended that future researchers consider using mixed method approaches to examine user experience outcomes of VR. For instance, follow-up interviews and focus groups could help identify aspects of the immersive VR interventions that most influenced user interest, usability, safety, and overall acceptability of VR interventions [71]. A more detailed consideration of economic factors (eg, cost of VR equipment, staff training, and maintenance) would be valuable for health care providers and policymakers considering implementation of these technologies, as it would help assess the feasibility of VR interventions, guide budgeting decisions, and support equitable access across clinical settings.

Presence, the subjective sense of being immersed in a virtual environment, may be critical for the effectiveness of VR-based interventions, but it remains underexplored. Increased presence positively predicts users’ reported usability of VR systems [42], and it is associated with heightened enjoyment and engagement [13,14]. However, our systematic review revealed that only 2 studies quantitatively assessed presence [51,52], one reporting it to be greater in the ADHD group, compared with the control group [52]. The other study described overall presence levels as high [51]. Given the potential for presence to enhance therapeutic outcomes [11], future studies ought to prioritize its assessment with standardized measures to better understand its role in the effectiveness of VR-based interventions for ADHD.

### Strengths and Limitations of the Systematic Review

This systematic review has several strengths and limitations. The review made use of a comprehensive search strategy, which included studies using a variety of research methodologies (both qualitative and quantitative), cognitive rehabilitation procedures that incorporated immersive VR for people of all ages with ADHD, and explored user experience outcomes alongside effectiveness outcomes. The review was also based on databases that comprised peer-reviewed publications. However, the review only included English-language research, which means studies in other languages would have been missed. In addition, the quality appraisal process is subjective. Therefore, though unlikely due to the use of multiple reviewers, researcher bias may have influenced the assessment reported in this review. Finally, this review did not include an appraisal of the cost-effectiveness of VR interventions, indicating a need for further research into their economic and practical feasibility in clinical settings.

### Conclusions

Immersive VR-based technology shows promise as a standalone or adjunctive treatment modality for rehabilitating cognitive abilities in people with ADHD. Effectiveness results indicate that immersive VR-based interventions can significantly improve attention, memory, and executive functions. Furthermore, VR has the potential to provide a safe, acceptable, and engaging treatment modality for clinicians to use with their child clients and adults who have ADHD. Nonetheless, caution is advised. It is premature to generalize these findings to wider populations due to the paucity of papers available and participants primarily being boys with ADHD. There is also a general absence of follow-up measurements. Therefore, future research using RCTs with mixed methods and longitudinal designs, such as follow-up interviews and focus groups, can verify the long-term effectiveness and user experience of immersive VR interventions in more diverse populations of people with ADHD.

## Supplementary material

10.2196/71963 Checklist 1 PRISMA 2020 checklist.

## Abbreviations

ADHD: attention-deficit/hyperactivity disorder
CPT: continuous performance test
HMD: head-mounted display
PRISMA: Preferred Reporting Items for Systematic Reviews and Meta-Analyses Checklist
RCT: randomized controlled trial
VR: virtual reality
